# Supplementary material for: TLR Responses in Preterm and Term Infant Cord Blood Mononuclear Cells
Source: Pathogens. 2023 Apr 14;12(4):596. doi: 10.3390/pathogens12040596 (PMC10145848; doi:10.3390/pathogens12040596)
Supplement: Supplementary file 1 [file pathogens-12-00596-s001.zip › Supplementary_Table_S1.pdf]

Supplementary Table S1: List of antibodies used for flow cytometric experiments.

**Antibody cocktail 1**	**Supplier**	**Dilution**
CD3-BUV395	BD Bioscience, San Diego, CA, USA	1/100
CD14-BUV805	BD Bioscience, San Diego, CA, USA	1/100
CD16-PacBlue	BD Bioscience, San Diego, CA, USA	1/400
CD56-BV711	BioLegend, San Diego, USA	1/50
CD19-BV785	BioLegend, San Diego, USA	1/400
CD11c-PerCP/Cy5.5	BD Bioscience, San Diego, CA, USA	1/200
CD123-PEcy7	BD Bioscience, San Diego, CA, USA	1/200
HLA-DR-APCH7	BD Bioscience, San Diego, CA, USA	1/200
Zombie NIR	BioLegend, San Diego, USA	1/800
TLR2-BV650	BD Bioscience, San Diego, CA, USA	1/100
TLR4-BV605	BD Bioscience, San Diego, CA, USA	1/200
**Antibody cocktail 2**	**Supplier**	**Dilution**
TLR3-APC	Miltenyi Biotec, Bergisch Gladbach, Germany	1/100
TLR7-FITC	Invitrogen, Thermofisher Scientific, CA, USA	1/100
TLR9-BV421	BioLegend, San Diego, USA	1/200
NF-KB-PE	Invitrogen, Thermofisher Scientific, CA, USA	1/100
