# Supplementary figures and images for: TLR Responses in Preterm and Term Infant Cord Blood Mononuclear Cells
Source: Pathogens. 2023 Apr 14;12(4):596. doi: 10.3390/pathogens12040596 (PMC10145848; doi:10.3390/pathogens12040596)

## Slide 1
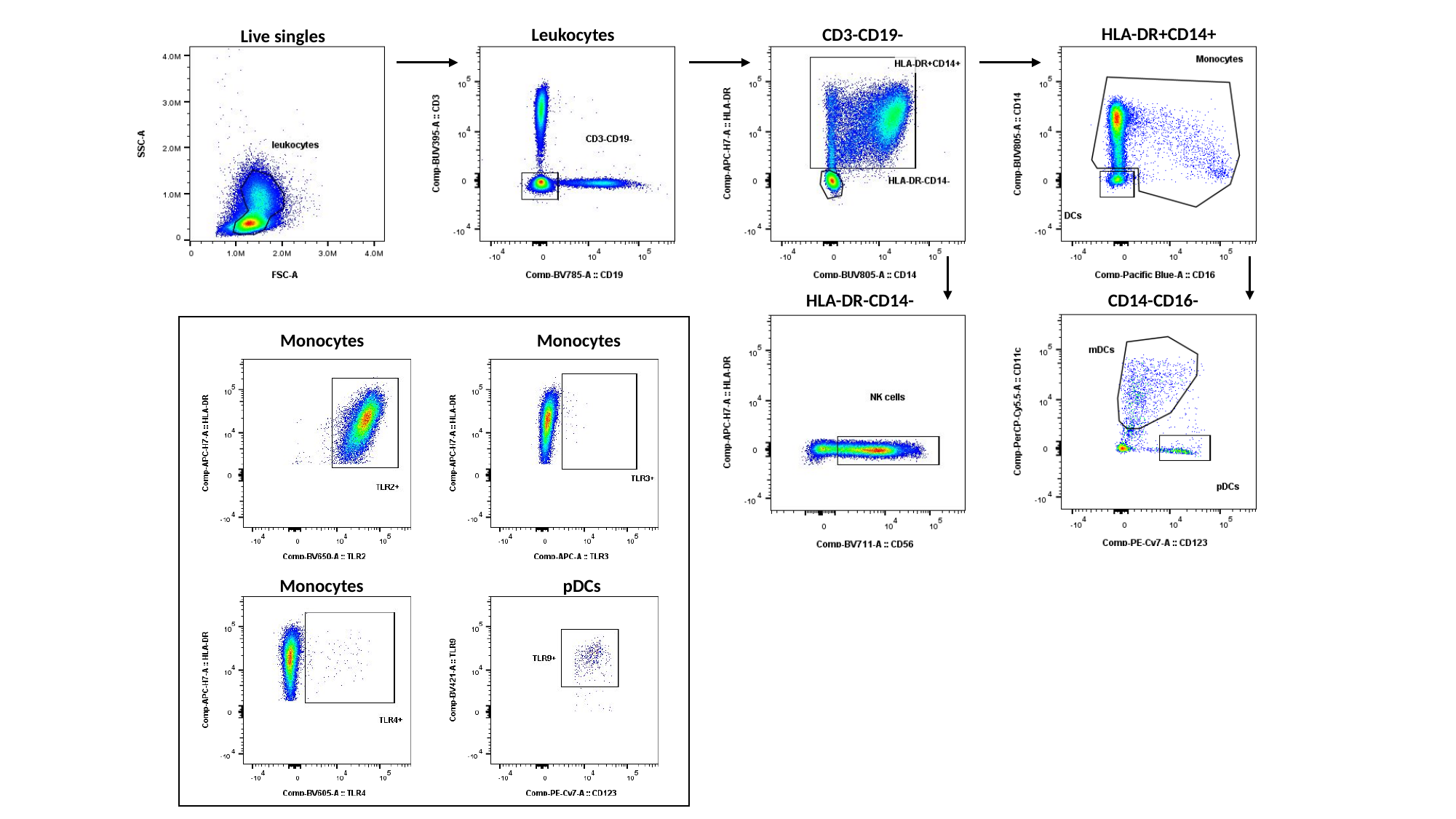

HLA-DR+CD14+
CD3-CD19-
Leukocytes
Live singles
HLA-DR-CD14-
CD14-CD16-
Monocytes
Monocytes
pDCs
Monocytes

Supplement: Supplementary file 1 [file pathogens-12-00596-s001.zip › Supplementary Figure S1.pptx]
